# GFRα1 Promotes Axon Regeneration after Peripheral Nerve Injury by Functioning as a Ligand

**DOI:** 10.1002/advs.202400812

**Published:** 2024-12-04

**Authors:** Tomoaki Suzuki, Ken Kadoya, Takeshi Endo, Miwako Yamasaki, Masahiko Watanabe, Norimasa Iwasaki

**Affiliations:** ^1^ Department of Orthopaedic Surgery Graduate School of Medicine Hokkaido University Sapporo Hokkaido 0608638 Japan; ^2^ Department of Anatomy Graduate School of Medicine, Hokkaido University Sapporo Hokkaido 0608638 Japan

**Keywords:** axon regeneration, GDNF family receptor, peripheral nerve injury, repair schwann cells

## Abstract

The neurotrophic factor, Glial cell line derived neurotrophi factor (GDNF), exerts a variety of biological effects through binding to its receptors, GDNF family receptor alpha‐1 (GFRα1), and RET. However, the existence of cells expressing GFRα1 but not RET raises the possibility that GFRα1 can function independently from RET. Here, it is shown that GFRα1 released from repair Schwann cells (RSCs) functions as a ligand in a GDNF‐RET‐independent manner to promote axon regeneration after peripheral nerve injury (PNI). Local administration of GFRα1 into injured nerve promoted axon regeneration, even more when combined with GDNF blockade. GFRα1 bound to a receptor complex consisting of NCAM and integrin α7β1 of dorsal root ganglion neurons in a GDNF‐RET independent manner. This is further confirmed by the Ret Y1062F knock‐in mice, which cannot transmit most of GDNF‐RET signaling. Finally, local administration of GFRα1 into injured sciatic nerve promoted functional recovery. These findings reveal a novel role of GFRα1 as a ligand, the molecular mechanism supporting axon regeneration by RSCs, and a novel therapy for peripheral nerve repair.

## Graphical abstract

Schwann cells increased the expression of GFRα1, which is known as a receptor for Glial cell line derived neurotrophi factor (GDNF), after peripheral nerve injury. GFRα1 functions as a ligand independently from the GDNF‐RET axis, binds to the complex of NCAM and integrin α7β1 on axons, and promotes axonal growth, indicating that GFRα1 is a novel axon regeneration factor.

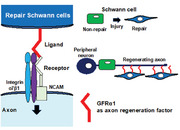

## 1 Introduction

Although the peripheral nervous system (PNS) is distinct from the central nervous system in that it can undergo regeneration, clinical outcomes after peripheral nerve injury (PNI) are generally poor, especially in cases of severe or proximal injuries and reconstruction cases.^[^ ^1^ ^]^ Accordingly, a novel treatment to promote regeneration after PNI needs to be developed. Following PNI, Schwann cells (SCs) undergo drastic changes, activate innate immune responses, secrete trophic factors, and remyelinate new axons,^[^ ^2^, ^3^, ^4^, ^5^ ^]^ indicating that SCs are pivotal in a variety of aspects of PNS repair. Recent studies categorized activated SCs after PNI as repair SCs (RSCs).^[^ ^6^, ^7^, ^8^, ^9^, ^10^, ^11^ ^]^ Glial cell line derived neurotrophi factor (GDNF) is a trophic factor secreted by RSCs, which regenerates axons and sustains their activated state after PNI.^[^ ^12^ ^]^ The expression of a receptor for GDNF, GDNF family receptor alpha‐1 (GFRα1), also increases in RSCs and axons after PNI to augment their autocrine and paracrine effects.^[^ ^13^, ^14^ ^]^ GFRα1 is a glycosyl phosphatidylinositol (GPI)‐anchored protein, lacks an intracellular domain, and requires co‐receptors, rearranged during transfection (RET) or neural cell adhesion molecule (NCAM) to transduce GDNF binding to an intracellular signal.^[^ ^15^, ^16^, ^17^, ^18^ ^]^ The GDNF signal cascade causes proliferation, migration, neurite outgrowth, axon guidance, and synapse formation.^[^ ^14^, ^15^ ^]^ Of note, GFRα1 is present not only on the cell surface but also in the extra‐cellular space following phospholipase‐mediated release from the GPI‐anchor.^[^ ^19^ ^]^ Soluble GFRα1 can still bind GDNF and stimulate RET to transmit the GDNF signal.^[^ ^20^ ^]^ Interestingly, GFRα1 expression is greater than RET in many tissues and cells,^[^ ^21^ ^]^ raising the possibility that GFRα1 also functions independently from the GDNF‐RET axis.^[^ ^22^ ^]^ GFRα1 alone can bind NCAM without forming a complex with RET or GDNF, leading to the downregulation of NCAM‐mediated cell adhesion.^[^ ^23^ ^]^ However, no other GDNF‐RET independent functions of GFRα1 have been identified to date, particularly those resulting in a ligand‐triggered intracellular signal.^[^ ^24^, ^25^ ^]^ In previous studies, exogenously applied GFRα1 always functioned as a soluble receptor mediating GDNF stimulation,^[^ ^21^, ^26^, ^27^ ^]^ excluding the possibility that GFRα1 functions as a ligand. Moreover, it should be noted that the function of GFRα1 has been investigated mainly with respect to its role in development using mice lacking the GFRα1, GDNF, or RET genes, which are necessary for live birth. Thus, the role of GFRα1 in the regeneration of the mature nervous system has been rarely analyzed. In the PNS, it is well known that SCs increase the expression of GFRα1 after PNI,^[^ ^28^ ^]^ yet the role of GFRα1 in the repair process remains obscure. Thus, the current study explored the role of GFRα1 in the repair process after PNI and demonstrated that GFRα1 binds to a complex of NCAM and integrin α7β1 as a ligand on adult DRG neurons in a GDNF‐RET independent manner, phosphorylates phosphoinositide 3‐kinase (PI3K), promotes axon regeneration and functional recovery after PNI. These findings reveal a novel GFRα1 function and indicate that the administration of GFRα1 has the potential as an effective therapy for PNI.

## 2 Result

### 2.1 SCs Express GFRα1 to Regenerate Axons after PNI

**Figure 1:**
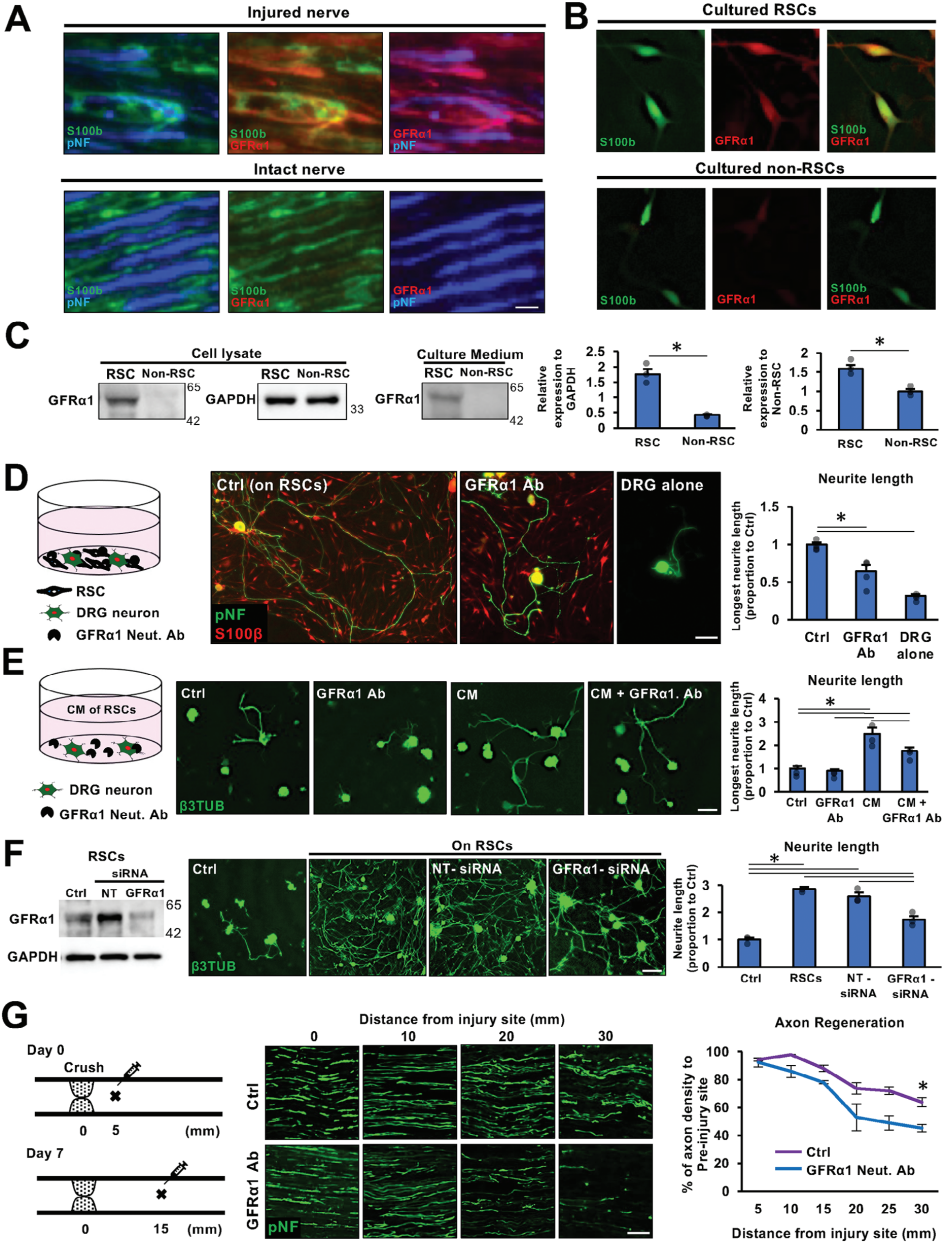
SCs express GFRα1 to regenerate axons after PNI. A) High magnification views of longitudinal sections of injured and intact sciatic nerves. The injured nerves are harvested 1 week after crush injury. Double immunolabeling of regenerating axons (pan‐neurofilament, pNF), SCs (S100b), or GFRα1. SCs expressing GFRα1 are closely associated with regenerating axons, whereas SCs in intact nerves do not express GFRα1. Scale bar, 10 µm. B) Representative images of cultured SCs. Non‐RSCs were prepared from intact nerves, and RSCs were from injured nerves receiving crush injuries 1 week before. Both SCs were stained after 24 h of culture. Immunoreactivity of GFRα1 was detected clearly in RSCs. Scale bar, 10 µm. C) Western blot detection of GFRα1 in SCs and their culture medium. Band intensities were quantified. Significantly more GFRα1 was detected in cell lysate and culture medium of RSCs than those of non‐RSCs. Individual dots indicate each data. ^*^ *P* < 0.05: Student's test. Error bars represent the SEM. N = 3/group. D) The co‐culture of RSCs and DRG neurons. Neurites and SCs were immuno‐labeled with β3tubulin (β3TUB) and S100β. RSCs stimulated neurite outgrowth of DRG neurons as expected, and the addition of GFRα1 neutralizing (Neut) antibody (Ab) significantly decreased their effect. Quantification was the longest neurite length normalized to the condition of co‐cultured with RSCs. Scale bar, 100 µm. Individual dots indicate each data. ^*^ *P* < 0.05: One‐way ANOVA with the Tukey–Kramer test. Error bars represent the SEM. N = 4/group. E) Culture of DRG neurons with conditioned media (CM) prepared from RSCs. The CM significantly promoted neurite outgrowth of DRG neurons immuno‐labeled with β3tubulin (β3TUB). The addition of GFRα1 Neut. Ab significantly reduced the effect of RSCs on neurite outgrowth. Scale bar, 100 µm. Individual dots indicate each data. ^*^ *P* < 0.05: One‐way ANOVA with the Tukey–Kramer test. Error bars represent the SEM. N = 3/group. F) Knock down of GFRα1 expression in RSCs by siRNA. Western blot of RSCs showed a partial reduction of GFRα1 by GFRα1 targeting siRNA but not non‐targeting (NT) siRNA. The knock down of GFRα1 expression in RSCs significantly reduced the neurite length. Scale bar, 100 µm. Individual dots indicate each data. ^*^ *P* < 0.05: One‐way ANOVA with the Tukey–Kramer test. Error bars represent the SEM. N = 3/group. G) Functional blocking of GFRα1 in injured nerves. GFRα1 Neut. Ab. was locally administered to injured rat sciatic nerve. Representative images of longitudinal sections immunolabeled for axons at the point of 0, 10, 20, and 30 mm distal to the injury site 2 weeks after injury. The Left is proximal. Scale bar, 10 µm. Quantification of the axon regeneration rate demonstrated that the administration of GFRα1 Neut. Ab significantly reduced axon regeneration after PNI. ^*^ *P* < 0.05: two‐way repeated measure ANOVA with the Bonferroni's test. Error bars represent the SEM. N = 3/group.

First, we investigated the expression of GFRα1 in SCs in intact nerves and RSCs.^[^ ^21^ ^]^ GFRα1 immunoreactivity was detected in RSCs associated with regenerated axons, whereas GFRα1 immunoreactivity was absent in SCs associated with intact axons (**Figure** **1**A). Similarly, cultured RSCs derived from injured nerves showed apparent GFRα1 immunoreactivity (Figure 1B). Cultured non‐RSCs from intact nerves demonstrated moderate GFRα1 immunoreactivity, which could be probably due to stress response by tissue dissociation procedure.^[^ ^29^ ^]^ Importantly, GFRα1 was also present in the culture medium of RSCs but not non‐RSCs (Figure 1C), indicating that GFRα1 was released from RSCs.^[^ ^20^ ^]^ To determine whether GFRα1 expression by RSCs contributes to axonal growth, we inhibited GFRα1 by the addition of a neutralizing antibody to the co‐culture of SCs and adult rat dorsal root ganglion (DRG) neurons. The inhibition of GFRα1 significantly shortened their neurite length by ≈40% (Figure 1D). The inhibition of GFRα1 in the conditioned medium with RSCs by the neutralizing antibody also significantly reduced the neurite length of DRG neurons (Figure 1E). Further, the knockdown of the expression of GFRα1 in RSCs by siRNA resulted in a significant decrease in neurite outgrowth (Figure 1F). Next, we inhibited GFRα1 in injured sciatic nerves by local administration of the GFRα1 neutralizing antibody (Figure 1G). The initial injection was at 5 mm from the injury site immediately after injury and the next one at 15 mm from the injury site 7 days after injury, followed by perfusion 1 week later. The subjects receiving the neutralizing antibody demonstrated significantly reduced axon regeneration compared to subjects receiving the control antibody (Figure 1G). Collectively, these findings indicate that RSCs increase the expression and secretion of GFRα1 after PNI and that GFRα1 contributes to the axon regeneration effect of RSCs.

### 2.2 GFRα1 Promotes Neurite Outgrowth Independently of GDNF‐RET Signaling

**Figure 2:**
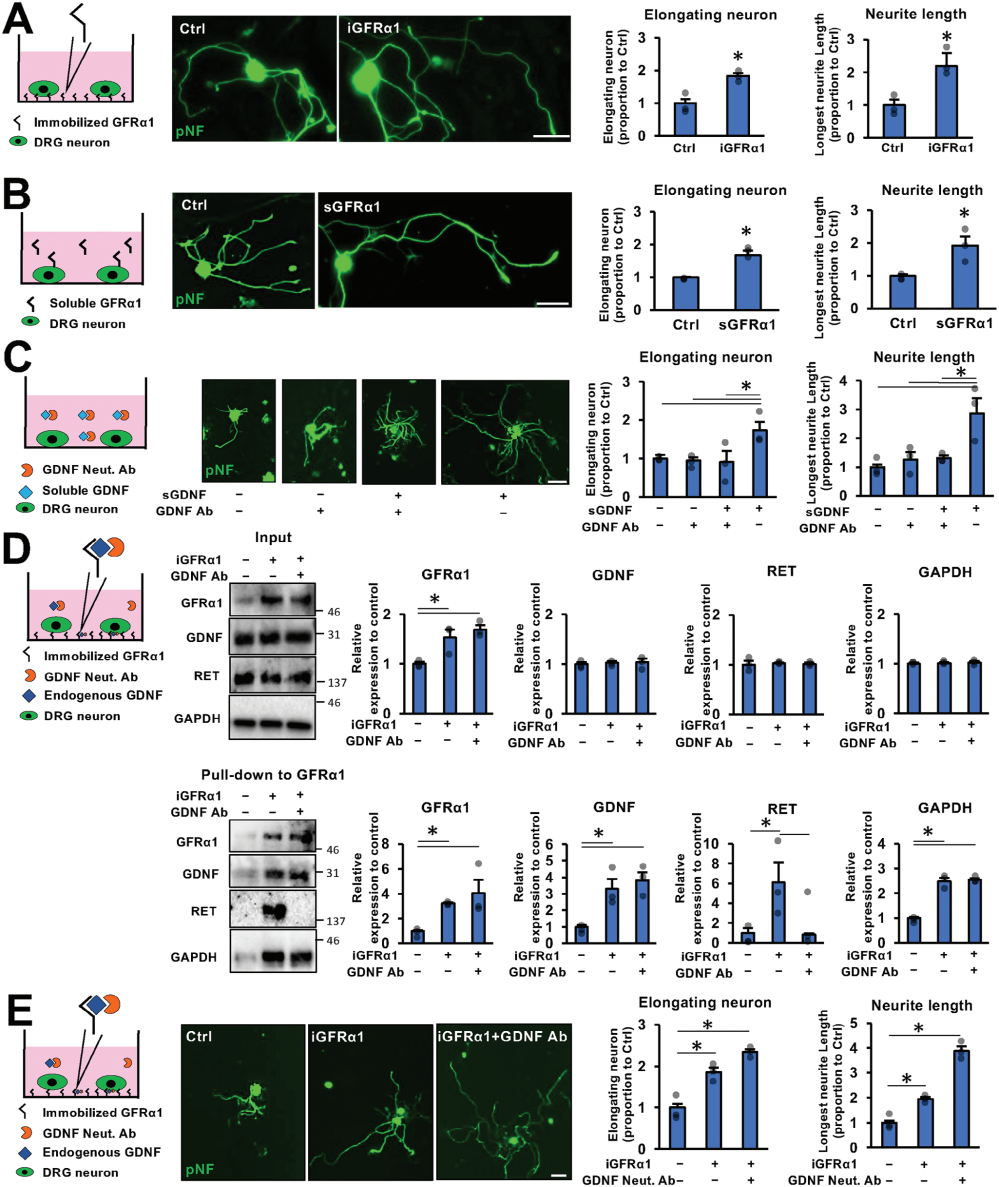
GFRα1 promotes neurite outgrowth of adult DRG neurons. A) DRG neurons were stimulated with immobilized GFRα1 (iGFRα1), resulting in the promotion of neurite outgrowth. Quantification of % of DRG neurons with elongating neurite and the average of the longest neurite length showed that iGFRα1 significantly enhanced neurite outgrowth. Individual dots indicate each data. ^*^ *P* < 0.05: Student's test. Error bars represent the SEM. N = 3/group. Scale bar, 100 µm. B) DRG neurons were stimulated with soluble GFRα1 (sGFRα1), resulting in the promotion of neurite outgrowth. Quantification of % of DRG neurons with elongating neurite and the average of the longest neurite length showed that sGFRα1 significantly promoted neurite outgrowth. Individual dots indicate each data. ^*^ *P*< 0.05: Student's test. Error bars represent the SEM. N = 3/group. Scale bar, 100 µm. C) The GDNF neut. Ab. abolishes the effect of exogenously applied GDNF on neurite outgrowth of DRG neurons. Quantification of % of DRG neurons with elongating neurite and the average of the longest neurite length. Values were normalized to control. Individual dots indicate each data. ^*^ *P* < 0.05: One‐way ANOVA with the Tukey–Kramer test. Error bars represent the SEM. N = 3/group. Scale bar, 100 µm. D) Western blotting of pull‐down assay to His‐tagged iGFRα1 in the lysate of DRG neurons. GDNF was identified regardless of the presence of GDNF Neut. Ab, but RET was not identified by the use of GDNF Neut. Ab. Band intensity was quantified by normalization to control. Error bars represent the SEM. ^*^ *P* < 0.05: One‐way ANOVA with the Tukey–Kramer test. N = 3/group. E) GDNF Neut. Ab. was added to the DRG culture to block the effect of endogenous GDNF. Quantification of % of DRG neurons with elongating neurite and the average of the longest neurite length demonstrated that GDNF Neut. Ab enhanced the effect of iGFRα1. Values were normalized to control. Individual dots indicate each data. Error bars represent the SEM. ^*^ *P* < 0.05: One‐way ANOVA with the Tukey–Kramer test. N = 3/group. Scale bar, 100 µm.

Since GFRα1 functions as a co‐receptor for GDNF together with RET, which transmits the GDNF signal intracellularly,^[^ ^8^, ^30^ ^]^ the observed axon impairment could result from the inhibition of GDNF‐ RET signaling. Thus, we aimed to determine the effect of exogenous administration of GFRα1 on neurite outgrowth of adult DRG neurons. Both the soluble and immobilized forms of GFRα1 significantly increased the proportion of elongating neurons and the length of the longest neurite compared to controls (**Figure** **2**A,B).

Because satellite cells are always present in cultured DRG neurons and could secrete a small amount of GDNF,^[^ ^31^ ^]^ it might form a complex with the administered GFRα1 and initiate GDNF‐ RET signaling. To circumvent this issue, we used a GDNF‐neutralizing antibody. It completely abolished the neurite‐promoting effect of GDNF (Figure 2C) by the blockade of the binding of GDNF with RET but not with GFRα1 (Figure 2D). The blockade of the GDNF‐RET signaling by this GDNF‐neutralizing antibody did not impair but further promoted the effect of GFRα1 on neurite outgrowth (Figure 2E), indicating its independency from the GDNF‐RET signaling.

**Figure 3:**
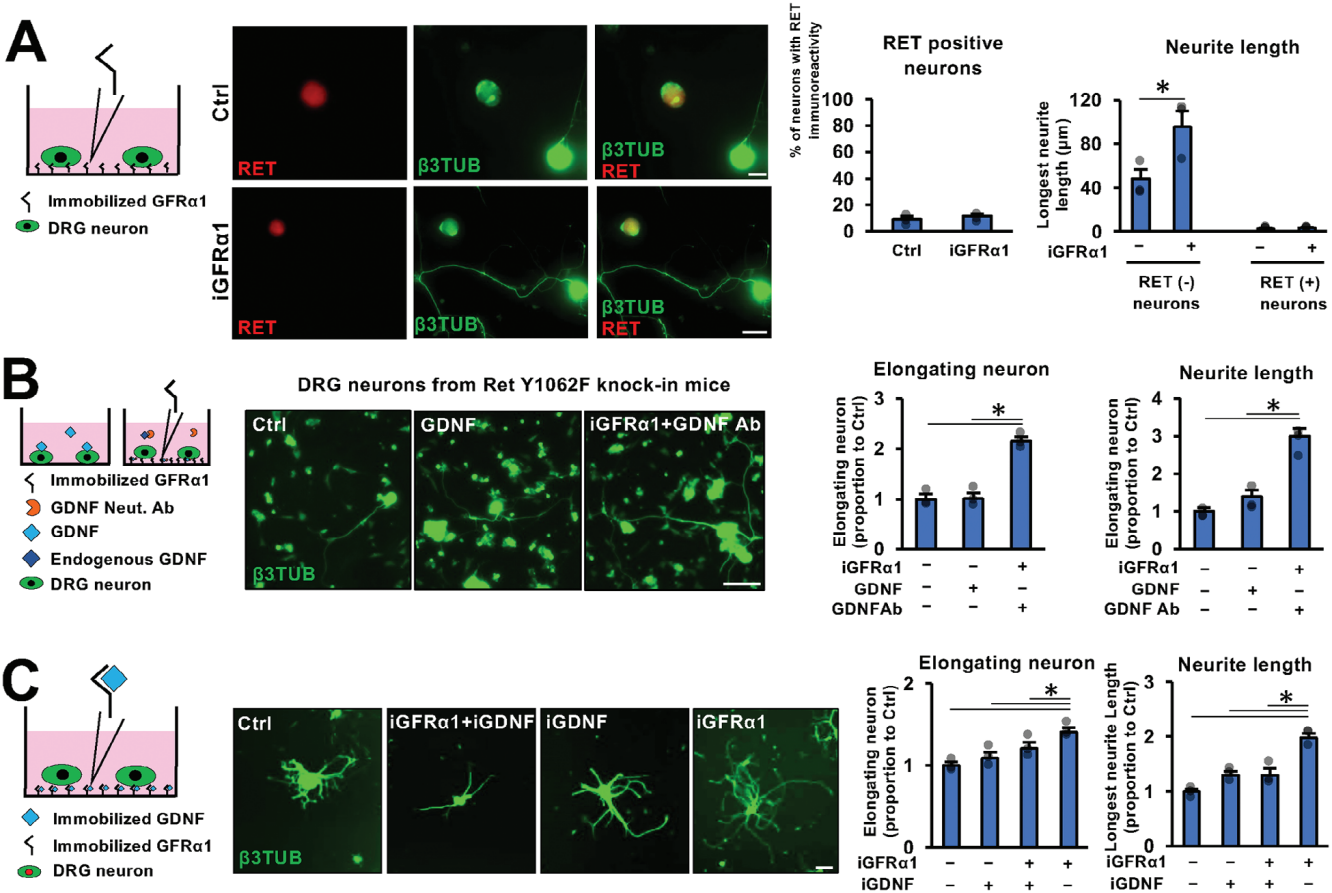
GFRα1 promotes neurite outgrowth of adult DRG neurons independently from the GDNF‐RET signal complex. A) Rat DRG neurons were stimulated with immobilized GFRα1 (iGFRα1) and immuno‐labeled with RET and β3tubulin (β3TUB). % of neurons showing immunoreactivity against RET and the average of the longest neurite length were quantified. iGFRα1 promoted neurite outgrowth of RET negative but not positive neurons. ^*^ *P* < 0.05: Student's test. Error bars represent the SEM. N = 3/group. Scale bar, 20 µm. B) Culture of DRG neuron from Ret Y1062F knock‐in mice. Neurons were stimulated with GDNF or iGFRα1. GDNF Neut. Ab. was added to the culture medium of the iGFRα1 condition to block the effect of endogenous GDNF. % of DRG neurons with elongating neurites and the average of the longest neurite length were quantified. Values were normalized to control. GDNF failed to promote neurite outgrowth, whereas iGFRα1 with GDNF Neut. Ab significantly increased neurite outgrowth. Individual dots indicate each data. ^*^ *P* < 0.05: One‐way ANOVA with the Tukey–Kramer test. Error bars represent the SEM. N = 3/group. Scale bar, 100 µm. C) Culture plates of DRG neurons were coated with GFRα1 (iGFRα1), followed by GDNF coating (immobilized GDNF, iGDNF). % of DRG neurons with elongating neurites and the average of the longest neurite length were quantified. Values were normalized to control. iGDNF abolished the effect of iGFRα1. Individual dots indicate each data. ^*^ *P* < 0.05: One‐way ANOVA with the Tukey–Kramer test. Error bars represent the SEM. N = 3/group. Scale bar, 100 µm.

Further, we analyzed the effect of GFRα1 on neurons not expressing RET, because only a subpopulation of DRG neurons express RET.^[^ ^32^ ^]^ The immunoreactivity against RET was detected in ≈10% of cultured adult DRG neurons, and the effect of GFRα1 on neurite outgrowth was observed only in DRG neurons, which showed no immunoreactivity against RET (**Figure** **3**A). Moreover, to confirm the independence from the GDNF‐RET signaling, we used the Ret Y1062F knock‐in mice in which tyrosine 1062 in Ret was replaced with phenylalanine. Since tyrosine 1062 in RET is a major binding site to initiate intra‐cellular signaling, this knock‐in mice cannot initiate most of GDNF‐ RET signaling pathways, such as the RAS/ERK, PI3K/AKT, and Jun‐associated N‐terminal kinase pathways.^[^ ^33^, ^34^ ^]^ Soluble GDNF did not increase the proportion of elongating neurons nor the length of the longest neurite at all (Figure 3B), whereas the combination of immobilized GFRα1 with the GDNF neutralizing antibody significantly increased both proportion of elongating neurons and the length of the longest neurite (Figure 3B), confirming the independency of the effect of GFRα1 on neurite outgrowth from the GDNF‐RET signaling.

Next, we examined how the effect of GFRα1 on neurite outgrowth was affected by the addition of GDNF. When culture plates with immobilized GFRα1 were coated with GDNF, the effect of GFRα1 was significantly attenuated (Figure 3C), suggesting that the GDNF‐GFRα1 complex is less effective in stimulating neurite outgrowth than GFRα1 alone. When soluble GDNF was added to the culture media, neurite outgrowth was stimulated (Figure S1) as expected,^[^ ^35^, ^36^, ^37^ ^]^ but the combination of soluble GDNF and immobilized GFRα1 did not produce additive effects on neurite outgrowth (Figure S1). These observations are likely due to all immobilized GFRα1 forming a complex with GDNF and the effect of GDNF being saturated.^[^ ^8^, ^35^ ^]^ In contrast, the combination of GFRα1 with inhibition of GDNF‐GFRα1‐RET signaling was the most effective in promoting neurite outgrowth under all conditions tested (Figure S1). These findings indicate that GFRα1 can function as a ligand to stimulate neurite outgrowth in adult DRG neurons independently of GDNF‐ RET signaling and that its neurite‐promoting effect is greater than the stimulation of GDNF‐GFRα1‐RET signaling.

Figure S1. The combination of GFRα1 with inhibition of GDNF had a greater neurite outgrowth effect than GDNF.

Quantification of % of DRG neurons with elongating neurite and the average of the longest neurite length. Values were normalized to control. GDNF failed to show an additive effect with iGFRα1 while blocking of GDNF with iGFRα1 demonstrated the greatest neurite outgrowth effect among all tested groups. Individual dots indicate each data. ^*^ *P* < 0.05: One‐way ANOVA with the Tukey–Kramer test. Error bars represent the SEM. N = 6/group.

### 2.3 GFRα1 Binds to a Complex of NCAM and Integrin α7β1 to Stimulate Neurite Elongation

**Figure 4:**
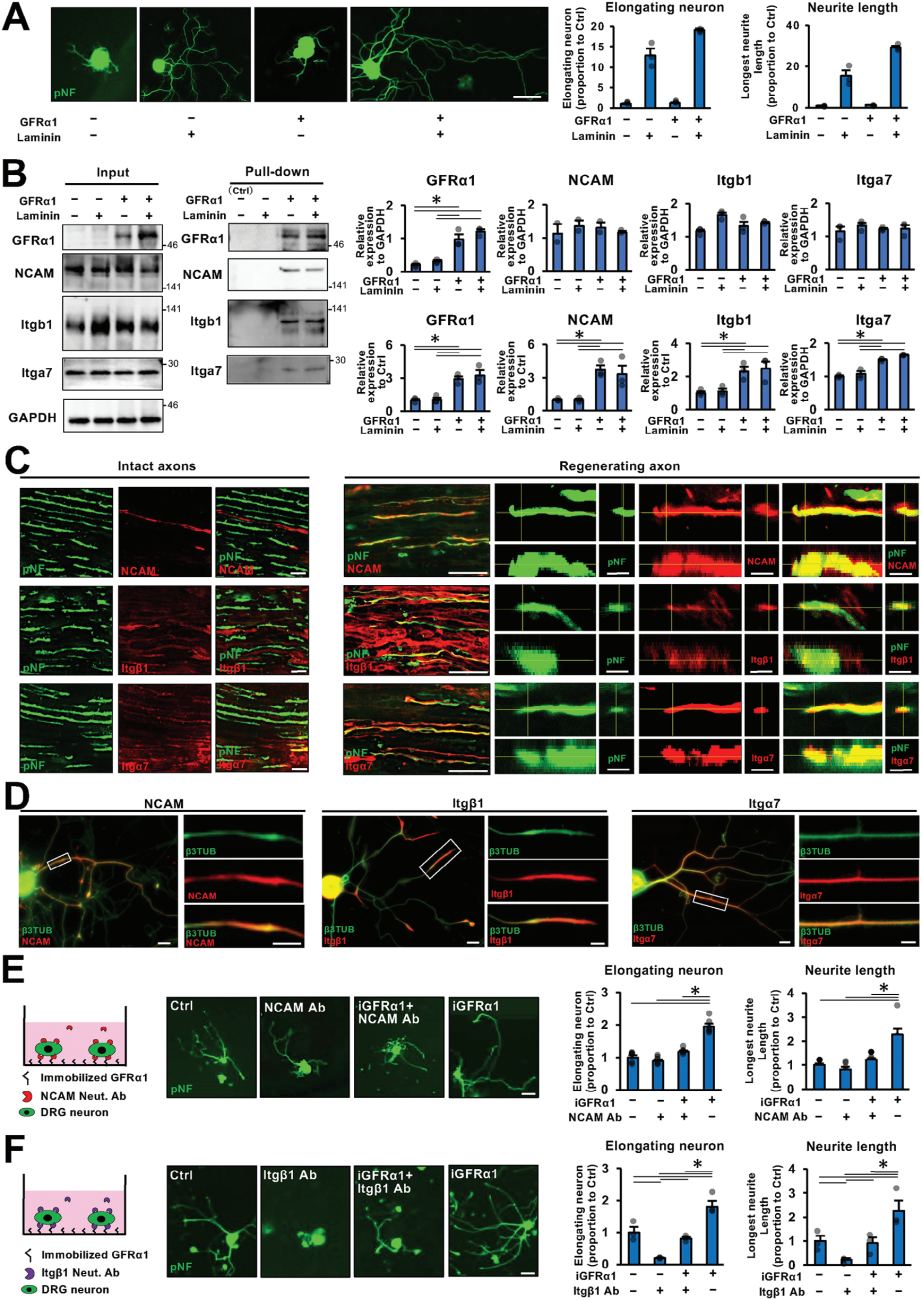
GFRα1 binds to a complex of NCAM and integrin α7β1 to promote neurite outgrowth. A) DRG neurons were stimulated by iGFRα1 with or without laminin coating. % of DRG neurons with elongating neurites and the average of the longest neurite length were quantified. The condition of no laminin coating was used as a control and values were normalized to the control. iGFRα1 failed to promote neurite outgrowth in the absence of laminin coating. Individual dots indicate each data. ^*^ *P* < 0.05: One‐way ANOVA with the Tukey–Kramer test. Error bars represent the SEM. N = 3/group. Scale bar, 50 µm. B) His‐tagged GFRα1 were pulled down from cultured DRG neurons with or without laminin coating and subjected to Western blotting. NCAM, integrin β1(itgβ1), and integrin α7 (itgα7) were detected in pull‐down samples when cultured with laminin. Band intensity was quantified by normalization to control. Error bars represent the SEM. ^*^ *P* < 0.05: One‐way ANOVA with the Tukey–Kramer test. N = 3/group. C) Immunolabeled images of regenerating and intact axons of longitudinal sections of rat sciatic nerves against axons (pNF), NCAM, Itgβ1, and Itgα7. Tips of regenerating axons express NCAM, Itgβ1, and Itgα7, whereas intact axons do not. Scale bars, 10 µm in intact axon images, 10 and 20 µm in lower and higher magnification images in regenerating axons. D) Representative images of cultured DRG neurons. Immunoreactivity of NCAM, Itgβ1, and Itgα7 was detected in neurites (pNF). Scale bar, 20 µm, and 10 µm in low and high magnification pictures, respectively. E) Cultured DRG neurons were stimulated with iGFRα1 and NCAM Neut. Ab. % of DRG neurons with elongating neurites and the average of the longest neurite length were quantified. Values were normalized to control. Neutralization of NCAM abolishes the effect of iGFRα1. Individual dots indicate each data. ^*^ *P* < 0.05: One‐way ANOVA with the Tukey–Kramer test. Error bars represent the SEM. N = 6/group. Scale bar, 100 µm. F) Cultured DRG neurons were stimulated with iGFRα1 and Itgβ1 Neut. Ab. % of DRG neurons with elongating neurites and the average of the longest neurite length were quantified. Values were normalized to control. Neutralization of Itgβ1 attenuated general neurite outgrowth and completely abolished the effect of iGFRa1. Individual dots indicate each data. ^*^ *P* < 0.05: One‐way ANOVA with the Tukey–Kramer test. Error bars represent the SEM. N = 3/group. Scale bar, 100 µm.

To identify a receptor for GFRα1 as a ligand, we investigated the laminin dependence of the GFRα1 effect on neurite outgrowth, since the extracellular matrix can regulate the affinity of ligand‐receptor binding.^[^ ^38^, ^39^ ^]^ Dramatically, GFRα1 failed to show an effect on neurite outgrowth in the absence of laminin coating (**Figure** **4**A), suggesting the possibility that the receptor for GFRα1 is activated by laminin. Accordingly, we performed a pull‐down assay and found that integrin α7β1, a receptor for laminin,^[^ ^27^ ^]^ was bound with GFRα1 (Figure 4B). We also observed that NCAM is bound with GFRα1 (Figure 4B).^[^ ^23^ ^]^ Importantly, the binding of GFRα1 to integrin α7β1 and NCAM was not attenuated in the absence of laminin, suggesting that integrin activation does not affect the involvement of NCAM with the receptor complex. In addition, an immunolabeling study showed that NCAM and integrin α7β1 were expressed in regenerating axons and cultured DRG neurons but not in intact axons (Figure 4C,D),^[^ ^40^, ^41^, ^42^ ^]^ indicating that they could act as GFRα1 receptors on axons. To determine the NCAM dependence of the effect of GFRα1 on neurite outgrowth, we inhibited NCAM function using a neutralizing antibody. The effect of GFRα1 was completely abolished following the inhibition of NCAM (Figure 4E). Next, integrin dependence was also determined by inhibiting integrin β1 with a neutralizing antibody. The inhibition of integrin β1 completely abolished the effect of GFRa1 and significantly attenuated general neurite outgrowth (Figure 4F). This general inhibition effect is probably because integrin β1 forms a molecular complex with not only integrin α7 but also other integrin members. Collectively, these findings indicate that as a ligand, GFRα1 binds to a complex of integrin α7β1 and NCAM to stimulate neurite outgrowth.

### 2.4 GFRα1 as a Ligand Activates PI3K but not ERK in Adult DRG Neurons

**Figure 5:**
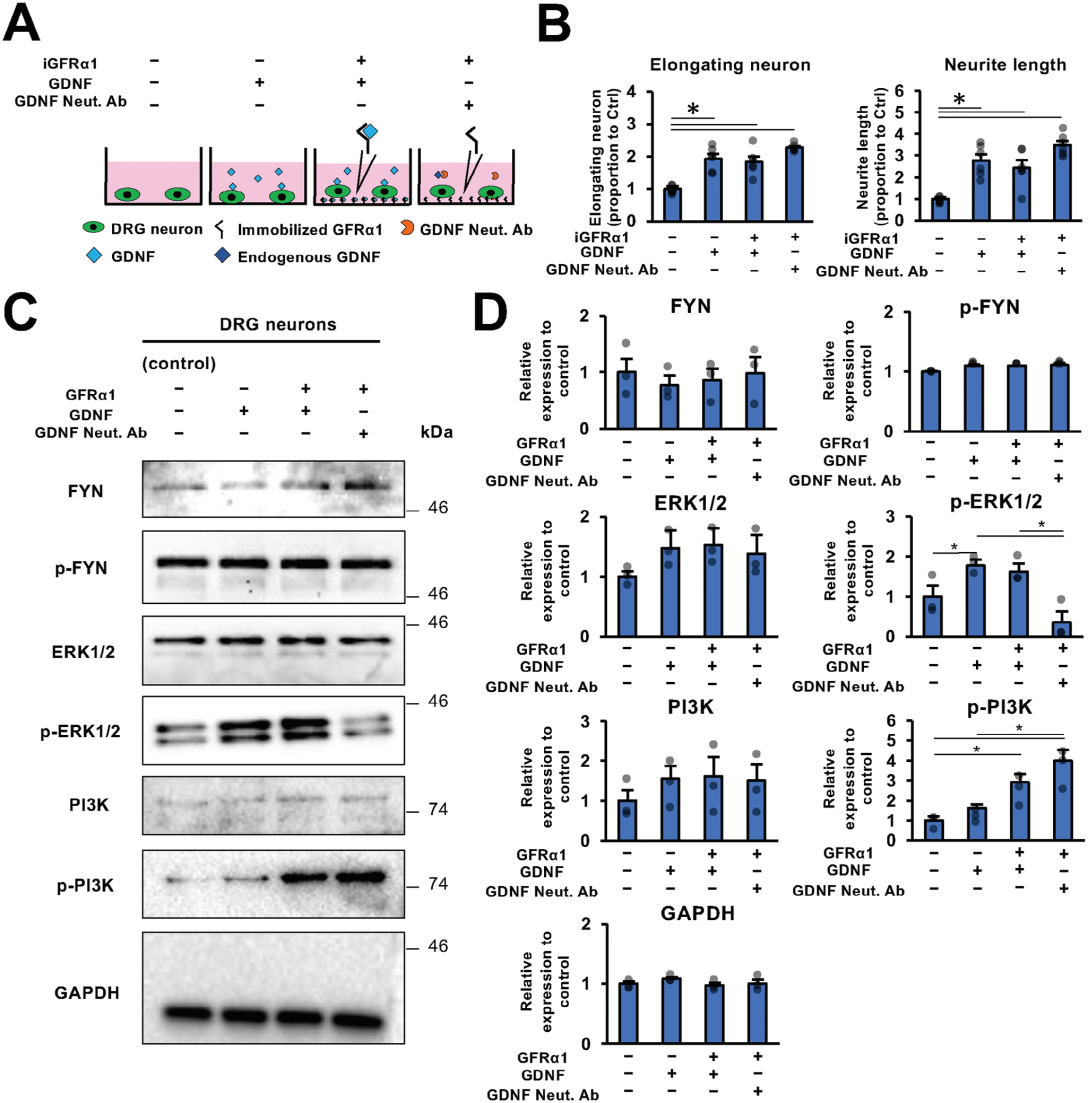
GFRα1 as a ligand activates PI3K but not ERK1/2 in adult DRG neurons. A) Scheme of the experiment to examine the combinatorial effect of GDNF and iGFRα1 on neurite outgrowth of DRG neurons. GDNF Neut. Ab was used to block endogenous GDNF. B) Quantification of % of DRG neurons with elongating neurite and the average of the longest neurite length. Values were normalized to control. Individual dots indicate each data. The addition of GDNF on iGFRα1 did not significantly promote neurite outgrowth. ^*^ *P* < 0.05: One‐way ANOVA with the Tukey–Kramer test. Error bars represent the SEM. N = 6/group. C,D) Representative images of Western blotting of DRG neurons with iGFRα1, GDNF, and GDNF Neut. Ab. The expression of FYN, p‐FYN, ERK1/2, p‐ERK1/2, PI3K, p‐PI3K, and GAPDH were analyzed. GAPDH was used as a housekeeping gene to normalize protein expression. Both iGFRα1 and GDNF did not activate FYN. iGFRα1activated PI3K but not ERK1/2, whereas GDNF activated ERK1/2 but not PI3K. Band intensities were calculated as relative to GAPDH. Individual dots indicate each data. ^*^ *P* < 0.05: One‐way ANOVA with the Tukey–Kramer test. Error bars represent the SEM. N = 3/group.

To clarify the intracellular signaling in neurons stimulated with GFRα1 as a ligand, Western blotting was performed on DRG neurons stimulated with iGFRα1 or GDNF (**Figure** **5**A,B). The expression of Fyn was investigated since it is known as a downstream signaling mediator of NCAM and integrin.^[^ ^43^, ^44^ ^]^ No upregulation or phosphorylation of Fyn was detected following stimulation with GFRα1 or GDNF (Figure 5C,D). ERK1/2 is known as a downstream signaling mediator of GDNF‐GFRα1‐RET,^[^ ^45^, ^46^ ^]^ and the expected increased phosphorylation in the presence of GDNF was observed, whereas GFRα1 in the presence of the GDNF neutralizing antibody did not enhance ERK1/2 phosphorylation (Figure 5C,D). Since PI3K is known to be a signaling mediating axon regeneration,^[^ ^47^, ^48^, ^49^ ^]^ we investigated its activation by GFRα1.^[^ ^50^ ^]^ It was enhanced to the greatest degree by GFRα1 with inhibition GDNF signaling, but GDNF stimulation alone did not significantly upregulate it (Figure 5C,D). These results indicate that GFRα1 enhances PI3K signaling when functioning as a ligand to promote neurite outgrowth, unlike when functioning as a receptor for GDNF.

### 2.5 Local Administration of GFRα1 Promotes Axon Regeneration after PNI

**Figure 6:**
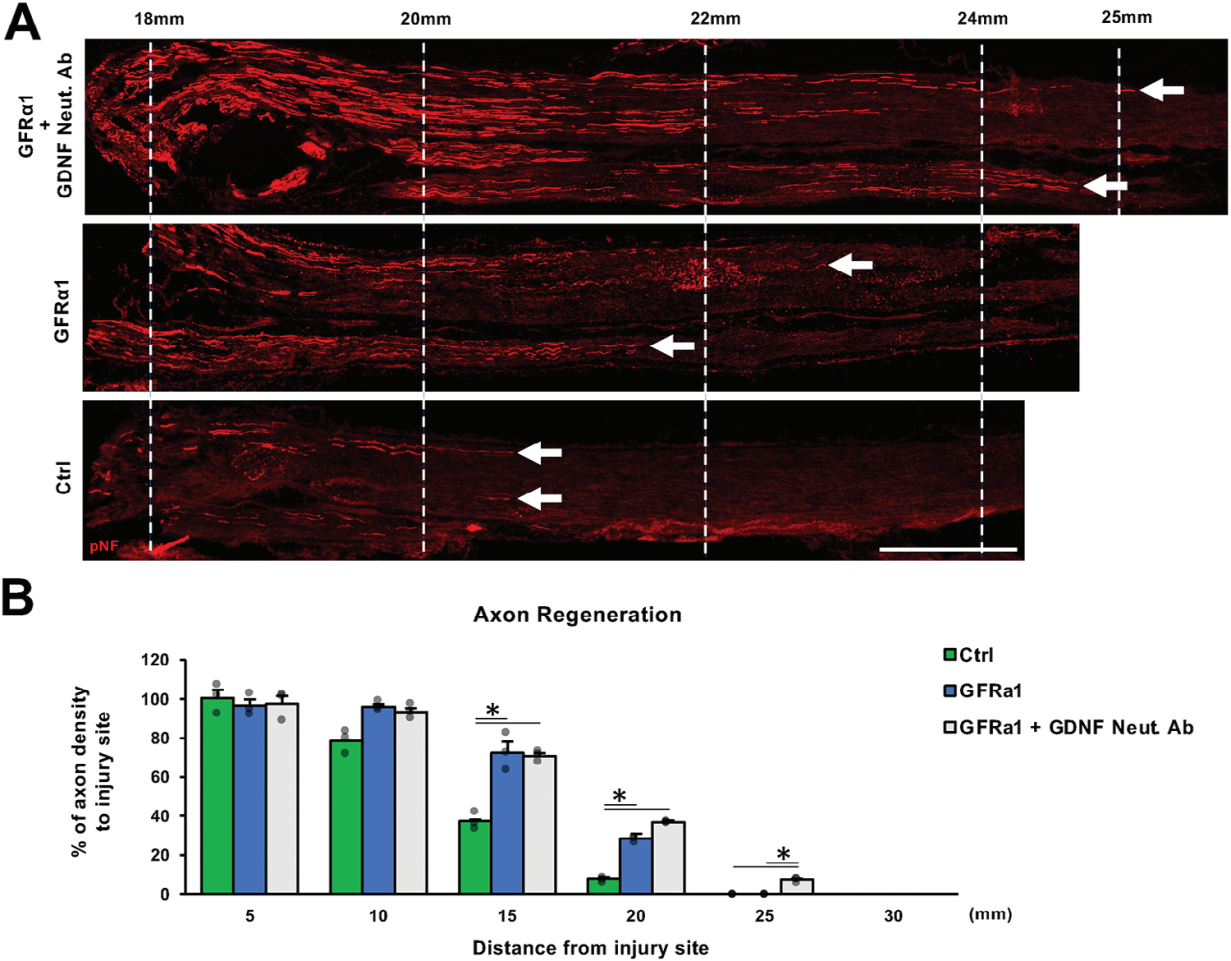
Local administration of GFRα1 promotes axon regeneration after PNI. A) Representative images of longitudinal sections immunolabeled for axons (pNF) at the point of 18–25 mm distal to the injury site 1 week after crush injuries and reagent injections. The left is proximal. Arrows indicate tips of regenerating axons. Scale bar, 1mm. B) Quantification of axon regeneration. GFRα1 with or without GDNF Neut. Ab induced significantly more axon regeneration than control at 15 and 20 mm distal to the injury site. Individual dots indicate each subject. ^*^ *P* < 0.05: One‐way ANOVA with the Tukey–Kramer test. Error bars represent the SEM. N = 3/group.

Based on the observation that GFRα1 can stimulate neurite outgrowth of adult DRG neurons, we aimed to determine whether GFRα1 can promote axon regeneration after PNI. Adult rats received crush injuries to sciatic nerves followed by local administration of GFRα1, GDNF neutralizing antibody, control protein, or control antibody, with subsequent perfusion 1 week later. The subjects receiving both GFRα1 and GDNF‐neutralizing antibodies demonstrated the greatest axon regeneration among the three groups (**Figure** **6**A,B). Subjects receiving GFRα1 alone exhibited statistically more axon regeneration than control subjects (Figure 6A,B). In summary, this result indicates that local administration of GFRα1 to injured nerves promotes axon regeneration and that axon regeneration can be further enhanced by concomitant inhibition of GDNF‐GFRα1‐RET signaling.

### 2.6 Local Administration of GFRα1 Promotes Functional Recovery after PNI

**Figure 7:**
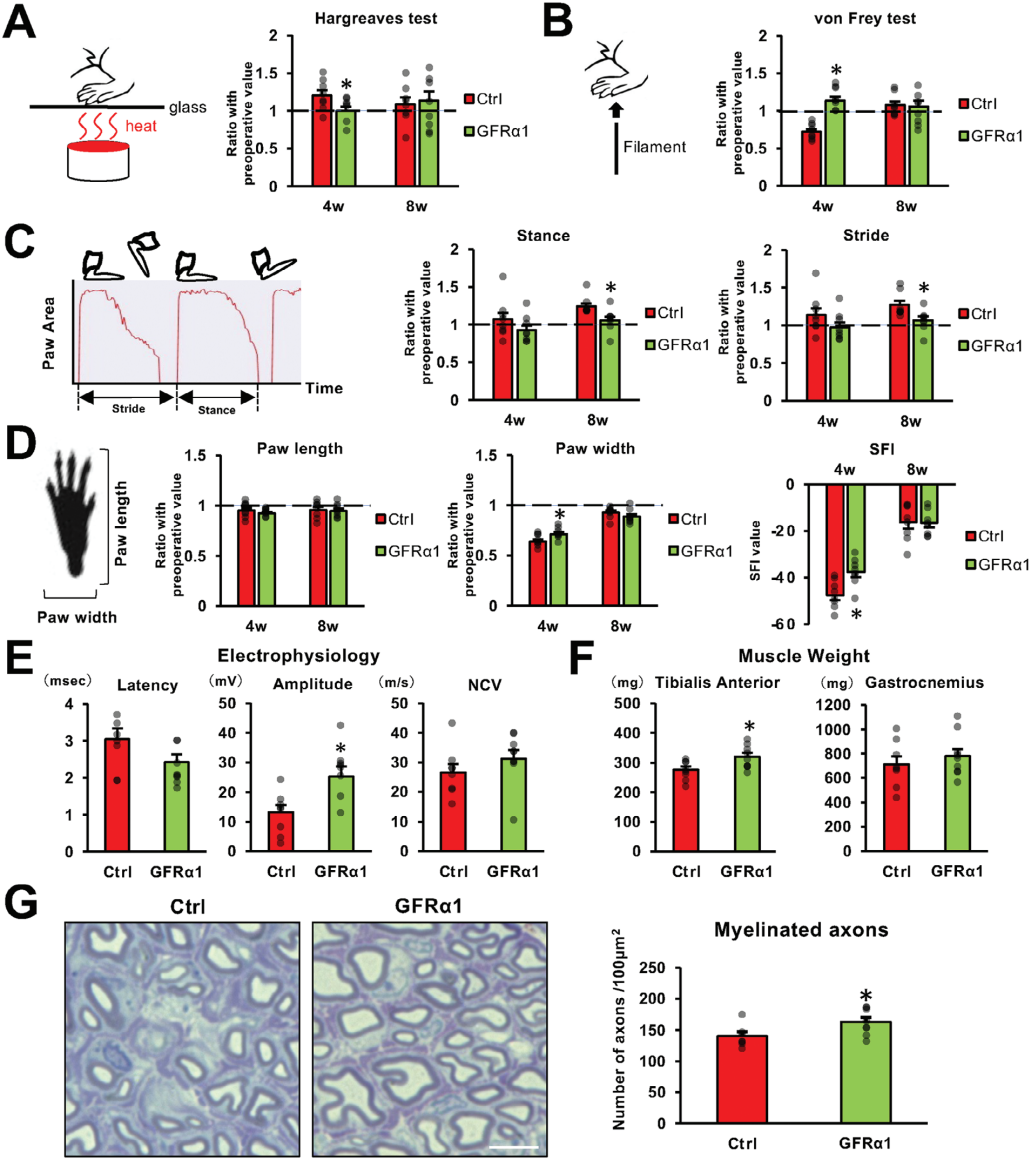
Local administration of GFRα1 promotes functional recovery after PNI. A–G) Subjects received local administrations of GFRα1 or control substance after crush injuries. N = 8/group. A) Left is the schema of the Hargreaves test. Right is the result normalized by pre‐injury value. GFRα1 treated subject demonstrated superior performance at 4 weeks after injury. Individual dots indicate each subject. ^*^ *P* < 0.05 to control: Student's test. Error bars represent the SEM. B) Left is a schema of the von Frey test. Right is the result normalized by pre‐injury value. GFRα1 treated subject demonstrated superior performance at 4 weeks after injury. ^*^ *P* < 0.05 to control: Student's test. Error bars represent the SEM. C) Left is a schema of stance and stride by DigiGait analysis. Middle is the result of stance normalized by pre‐injury value. Right is the result of stride normalized by pre‐injury value. GFRα1 treated subject demonstrated superior performance at 8 weeks after injury. ^*^ *P* < 0.05 to control: Student's test. Error bars represent the SEM. D) Left is a schema of paw length and width. Two in the middle are the results of paw length and paw width normalized by pre‐injury value. Right is the result of the sciatic functional index (SFI) calculated by paw length and paw width. GFRα1 treated subjects of paw width and SFI demonstrated superior performance at 4 weeks after injury. ^*^ *P* < 0.05 to control: Student's test. Error bars represent the SEM. E) The result of latency, amplitude, and NCV in electrophysiological experiment. ^*^ *P* < 0.05 to control: Student's test. Error bars represent the SEM. F) The result of tibialis anterior and gastrocnemius muscle weights. ^*^ *P* < 0.05 to control: Student's test. Error bars represent the SEM. G) The left is a representative image of remyelinated axons. Right is a quantification of remyelinated axons staining with toluidine blue. ^*^ *P* < 0.05 to control: Student's test. Error bars represent the SEM. Scale bar, 5 µm.

Next, we investigated the therapeutic potential of GFRα1 by evaluating functional recovery after PNI. Immediately after crush injuries to rat sciatic nerves, GFRα1 was locally injected into the injured nerves as in the preceding experiment, followed by 8 weeks of observation. In the Hargreaves test, control subjects demonstrated prolonged reaction times in the affected paw at 4 weeks after injury, whereas subjects in the GFRα1 group exhibited reaction times comparable to the pre‐injury state (**Figure** **7**A). At 8 weeks after injury, subjects in both groups demonstrated almost normal reaction times. In the von Frey test, at 4 weeks after injury, the reaction times in GFRα1 treated subjects were closer to the pre‐injury state than the control subjects (Figure 7B). At 8 weeks after injury, there was no significant difference in reaction times between the two groups (Figure 7B). These results indicate that the sensory functions evaluated with heat and contact stimulation were impaired at 4 weeks after injury but recovered at 8 weeks after injury in the rat sciatic nerve crush model and that the application of GFRα1 accelerated the recovery of sensory function. Gait analysis showed that there was no statistical difference in stance and stride time between both groups at 4 weeks after injury (Figure 7C). At 8 weeks after injury, the stance and stride times of the subjects receiving GFRα1 were closer to the pre‐injury state than the control subjects (Figure 7C). Regarding paw function, paw length did not change much after injury (Figure 7D). In contrast, paw width decreased at 4 weeks after injury and recovered at 8 weeks after injury, and paw width in the GFRα1 treated subjects was significantly closer to the pre‐injury state at 4 weeks after injury. Calculation of the sciatic functional index (SFI) showed that the GFRα1 treated subjects also improved at 4 weeks. These results indicate that GFRα1 administration promotes recovery of gait function after PNI. Analysis of the electrophysiological properties of injured sciatic nerves indicated that there was no significant difference in nerve conduction velocity (NCV) between the two groups. However, a tendency for shorter latency (p = 0.1) was detected in the subjects receiving GFRα1. The amplitude of action potentials in the tibialis anterior (TA) of subjects receiving GFRα1 was about twice as high as the control (Figure 7E). Furthermore, analysis of TA muscle weights indicated that subjects receiving GFRα1 exhibited significantly heavier TA muscles than the control subjects (Figure 7F), suggesting that the GFRα1 treatment suppressed muscle atrophy. Lastly, quantification of the number of myelinated axons at the most distal site of sciatic nerves showed that the GFRα1 group exhibited significantly more myelinated axons than the control group (Figure 7G), indicating that GFRα1 administration promoted axon regeneration and remyelination. Collectively, these findings indicate that the local administration of GFRα1 promotes functional recovery after PNI.

## 3 Discussion

The current study demonstrates that GFRα1 functions as a ligand independently of GDNF‐RET signaling by binding to a complex of NCAM and integrin α7β1 on injured axons to promote axonal growth via enhancement of PI3K signaling. In addition, local administration of GFRα1 to injured nerves accelerated axon regeneration and improved functional recovery, indicating that GFRα1 has therapeutic potential for PNI.

The fact that GFRα1 acts as a GDNF‐RET‐independent ligand partially explains why the distribution of GFRα1‐expressing cells is greater than that of RET‐expressing cells.^[^ ^36^ ^]^ The observed independence of GFRα1 from GDNF‐RET signaling in adult DRG neurons is in sharp contrast to a previous study showing that the effect of GFRα1 on neurite outgrowth in developing neurons is completely GDNF dependent.^[^ ^51^ ^]^ The main reason behind this discrepancy is likely to be the developmental stages of the analyzed neurons. The current study used mature neurons, whose phenotype is distinctly different from the developing neurons used in previous studies. For instance, the intrinsic growth of mature neurons is modest compared to that of developing neurons.^[^ ^52^ ^]^ Furthermore, mature neurons require support from SCs, whereas developing neurons do not,^[^ ^53^, ^54^ ^]^ as well as a permissive substrate for axonal growth in the PNS.^[^ ^55^, ^56^ ^]^ Although the current study only examined the independency of the effect of GFRα1 from GDNF‐RET signaling, the interaction with other GDNF family ligands, such as neurturin and artemin, also needs to be considered. They preferentially bind to GFRα2 and GFRα3 respectively, but, at the same time, they could interact with GFRα1,^[^ ^57^ ^]^ raising a possibility that signaling molecules other than GDNF might be involved with a complex consisting of GFRα1, NCAM, and integrin α7β1. Since research into the GFRα receptor family using the mature nervous system is still limited, further studies using mature neurons could elucidate new aspects of GFRα receptor functions.

When GFRα1 functions as a ligand, it binds a complex consisting of NCAM and integrin α7β1 independently from GDNF‐RET binding. NCAM is a strong candidate as the direct binding site of the receptor complex because it can directly bind GFRα1 in the absence of GDNF.^[^ ^21^ ^]^ Regarding integrin, it is suggested that integrin β1 can also bind to GFRα1.^[^ ^58^ ^]^ Therefore, GFRα1 may bind to the receptor complex at either the NCAM or integrin α7β1 sites. Interestingly, the binding of GFRα1 to the receptor complex is independent of laminin but its neurite outgrowth effect is laminin‐dependent. Accordingly, we speculate the binding mode of GFRα1 as a ligand is the following fashion. The binding of GFRα1 to NCAM or integrin α7β1 enables the formation of a complex consisting of GFRα1, integrin α7β1, and NCAM, and then activation of integrin by laminin subsequently initiates intracellular signaling through integrin or NCAM. Certainly, it is possible that other unidentified molecules, in addition to integrin α7β1 and NCAM, might be components of the receptor complex and contribute to mediating the effects of GFRα1. Elucidation of these molecular details will contribute to understanding how GDNF family receptors are involved in the maintenance and repair processes of the mature nervous system.

When adult DRG neurons were stimulated by GFRα1 in the absence of GDNF, PI3K was enhanced while ERK was not. In contrast, GDNF stimulation enhanced ERK but not PI3K. Since crosstalk between ERK and PI3K does not occur in neurons,^[^ ^59^ ^]^ this result suggests that GFRα1 ligand function elicits an intracellular signal that is distinct from its effects as a receptor for GDNF. Although both PI3K and ERK are major intracellular signals mediating axonal regeneration,^[^ ^60^ ^]^ one study reported that enhancement of PI3K induced more axon regeneration than enhancement of ERK in the PNS,^[^ ^61^ ^]^ which supports the current findings. Stimulation of adult DRG neurons by GFRα1 as a ligand did not increase expression of Fyn nor induced its phosphorylation, which is known as a direct downstream signal of NCAM and integrin^[^ ^62^ ^]^ as well as an upstream signal of PI3K.^[^ ^63^, ^64^, ^65^ ^]^ Therefore, an unidentified co‐receptor might exist as a receptor complex with NCAM and integrin and transmit signals through its intracellular domain. Similarly, NCAM or integrin might transmit their signals through an unknown intracellular molecule other than Fyn.

Although GFRα1 can stimulate neurite outgrowth in immobilized form as well as soluble form equivalently (Figure 2A,B), the current study employed the immobilized form in most in vitro experiments. GFRα1 is always located at the cell surface as a receptor for GDNF,^[^ ^17^ ^]^ regenerating axons are always closely associated with RSCs,^[^ ^66^ ^]^ and the effect of cell‐contact factors on SCs in neurite outgrowth is comparable to that of secreted factors.^[^ ^10^, ^67^ ^]^ Further, the actual amount of released GFRα1 from RSCs in vivo remains unclear. Therefore, it appears reasonable to analyze its immobilized form rather than soluble form to simulate in vivo conditions as the initial investigation of the molecular mechanisms of GFRα1 as a ligand.

Because SCs increase GFRα1 expression after PNI, the concentration of GFRα1 in injured nerves might have reached levels that produce a maximal effect. However, local administration of GFRα1 into injured nerves promoted axon regeneration, indicating that the local concentration of GFRα1 in injured nerves was insufficient to produce a maximal effect on stimulating axonal growth. The axon‐promoting effect of GFRα1 could be mediated by a mechanism involving it acting as a receptor for GDNF as well as a ligand. Because the effect of GDNF on neurite outgrowth is saturated at a low dose,^[^ ^35^ ^]^ no studies have reported that local administration of GDNF promotes axon regeneration, besides a study using long‐term continuous supply to the nerve defects with materials such as gel and conduit,^[^ ^68^ ^]^ it appears that local administration of GFRα1 stimulated axons by functioning as a ligand rather than as a GDNF receptor. Because GDNF promotes axon regeneration and maintains SCs in an activated state,^[^ ^45^, ^69^ ^]^ inhibition of GDNF by application of a neutralizing antibody could hamper axon regeneration after PNI. However, GDNF inhibition combined with GFRα1 administration further promoted axon regeneration. This indicates the ligand effect of GFRα1 on promoting axon regeneration is potent, as shown in the in vitro studies.

A previous study generated mutant mice with the GFRα1 gene deleted in cells not expressing RET to determine whether RET‐independent GFRα1 contributes to axon regeneration after PNI.^[^ ^21^ ^]^ Based on the result showing that the extent of axon regeneration in the mutant mice was comparable to that of wild type, this study concluded that RET‐independent GFRα1 was not required for axon regeneration after PNI. This appears to be contradictory to the findings of the current study. However, the expression of GFRα1 in SCs from the mutant mice is unaffected because SCs express RET. Therefore, the amount of GFRα1 in the region of Wallerian degeneration was likely unchanged, explaining why reduced axon regeneration was not observed in these mutant mice.

Although GFRα1 as a ligand requires laminin coating to induce neurite outgrowth of cultured adult DRG neurons, co‐administration of laminin with GFRα1 in injured nerve was not necessary for promoting axon regeneration after PNI. This is because laminin is abundant in injured nerves as it is the main extra‐cellular matrix component of the basement membrane in the endoneurium.^[^ ^70^ ^]^ Further, regenerating axons always accompany SCs to secrete laminin.^[^ ^55^, ^66^ ^]^ Accordingly, administration of GFRα1 without laminin into injured nerves induced axon regeneration.

PNI at proximal sites, such as the brachial plexus, always results in poor motor function.^[^ ^71^, ^72^, ^73^ ^]^ This is because denervated muscles lose their sensitivity to reinnervation if the timing of reinnervation passes the critical period.^[^ ^74^, ^75^ ^]^ Therefore, an effective therapy to accelerate the rate of axon regeneration needs to be developed. The PNI model used in the current study was a crush injury in rat sciatic nerve, in which all axons eventually regenerate and impaired functions recover greatly,^[^ ^76^ ^]^ unlike other model injuries such as transection and reconstruction with a conduit. Therefore, promoting axon regeneration at 2 weeks and 8 weeks after injury in this model means that the rate of axon regeneration increased, and its effect was sustained. Regarding functional recovery, several parameters such as sensory functions, paw length, and paw width in control subjects demonstrated almost full recovery to the pre‐injure state at 8 weeks after injury. However, at 4 weeks, subjects treated with GFRα1 showed better performance in sensory functions and paw width, also supporting the fact that GFRα1 accelerates recovery after PNI. Overall, the therapeutic effect of GFRα1 was detected but its magnitude was not substantial. This could be due to the characteristics of the crush model, which shows great spontaneous recovery,^[^ ^76^ ^]^ or the limit of the therapeutic efficacy of GFRα1. The additional evaluation of GFRα1 using other PNI models such as transection and reconstruction with a conduit is necessary to clarify its therapeutic potential. When considering its clinical translation, its local administration with direct vision during surgery or through a percutaneous rout under ultrasonic guidance could be applied.^[^ ^77^ ^]^ In addition to the local administration, GFRα1 can be utilized in artificial nerve conduits by surface coating, since immobilized GFRα1 also effectively induces axon regeneration.

In summary, GFRα1 not only acts as a receptor for GDNF but also functions as a ligand in a GDNF‐RET independent fashion to promote axonal growth of adult peripheral neurons, thus expanding understanding of the functions of GFRα receptors. The provision of GFRα1 to adult peripheral neurons represents a new treatment option for conditions requiring axon regeneration.

## 4 Experimental Section

### Animal

Adult LEWIS male and female rats weighing from 160 to 220 g (8–16 weeks old, Charles River Laboratories Japan, Inc.) were used. Ret Y1062F knock‐in mice (RBRC06249) were provided by RIKEN BRC through the National BioResource Project of the MEXT/AMED, Japan. The mice were produced and genotyped as described previously.^[^ ^33^ ^]^ This study was performed in line with the principles of the Declaration of Helsinki and carried out in accordance with the recommendations of the local ethical committee of Hokkaido University (17‐0071, 22‐0090). Animals took food and water freely throughout the study. For animal anesthesia, a mixture of ketamine (100 mg kg^−1^, KETALAR, Daiichi Sankyo Propharma Corporation, Tokyo, Japan) and medetomidine (0.5 mg kg^−1^, DOMITOR, Orion Corporation, Espoo, Finland) was administered by intramuscular injection. When euthanizing subjects humanely, an overdose of a mixture of ketamine and medetomidine was intraperitoneally injected.

### SC Preparation

SCs were prepared from rat sciatic nerves. SCs from intact nerves were defined as non‐RSCs and from nerves receiving crush injuries 1 week before preparation were defined as RSCs. SC preparation was performed according to the modification of a previous protocol.^[^ ^78^ ^]^ Nerves were dissected from the sciatic notch to the end of the femur, cut into 1–2 mm pieces using micro‐scissors after removal of the epineurium, and digested in the medium containing enzyme, 1% collagenase I (Sigma–Aldrich, St. Louis, MO) and 0.125% trypsin in Dulbecco's modified Eagle Medium (DMEM)/Ham's F‐12 (Wako, Osaka, Japan). After incubation for 1 h at 37 °C, 10% fetal bovine serum (FBS, Sigma–Aldrich, St. Louis, MO) in DMEM to stop the enzymatic reaction. After centrifuge and remove of the supernatant, nerve pieces were dissected mechanically by triturating with a pipette 30 times in SC culture medium, which consisted of DMEM/Ham's F‐12 supplemented with 10% FBS, 1% GlutaMAX (Thermo Fisher Scientific, Waltham, MA), and 1% penicillin‐streptomycin (PS, Thermo Fisher Scientific, Waltham, MA). The cell suspension was filtered through a 40 µm cell strainer to remove the pieces of tissue and plated on the uncoated flask for 30 min to remove the fibroblasts. The cell viability was assessed with trypan blue (Life Technologies, Grand Island, NY). Cells were seeded in flasks coated with poly‐L‐lysine (PLL, Sigma–Aldrich, St. Louis, MO) and laminin (Sigma–Aldrich, St. Louis, MO), and incubated for 1day before the usage for the analysis. To detect GFRα1 in the culture medium, it was centrifuged at 3000 g for 10 min, and the supernatant was used for Western blotting. To detect GFRα1 in SCs, SCs were washed with PBS 3 times and subject to a lysis buffer described below.

### Neurite Outgrowth Assay—DRG Neuron Culture

Rat adult DRG neurons were prepared as described previously.^[^ ^79^ ^]^ Dissected DRGs were enzymatically digested in DMEM/Ham's F‐12 with 0.5% collagenase XI (Sigma–Aldrich, St. Louis, MO) at 37 °C for 1 h. After removing digestion media, 1 mL DRG medium consisting of DMEM/Ham's F12 with 2% B27 supplement (Thermo Fisher Scientific, Waltham, MA), 1% P/S, and 1% GlutaMAX was added. The DRG pieces were gently triturated with a 1 mL pipette. 1.0 × 10^4^ DRG neurons cm^−2^ were seeded on the 48‐well plate which was coated with PLL and laminin in the DRG media. 48 h later, cells were fixed with 4% paraformaldehyde (PFA, Nacalai Tesque Inc., Kyoto, Japan) in 0.1 m phosphate buffer. Neurites were immunolabeled with pan neurofilament (pNF) described below. DRG neurons of Ret Y1062F knock‐in mice were prepared from postnatal day 1 because most of them died soon after birth. The neuron preparation procedure was the same as the case of adult rat DRG neurons.

### Neurite Outgrowth Assay—Quantification of Neurite Length

Immunolabeled neurite was quantified as described previously.^[^ ^67^ ^]^ Images were taken by an all‐in‐one fluorescent microscope (BZ‐X710, Keyence, Osaka, Japan) using a 20x objective lens. Neurites were traced and measured by Image J^[^ ^80^ ^]^ with plugin software, Neuron J. To avoid the effect of neuron‐neuron interaction, if their neurites touched the neurites of other neurons, these neurons were excluded from the analysis. 50 µm or longer neurite was defined as an elongating neurite.^[^ ^81^ ^]^ At least 50 neurons were randomly selected per well, and the % of neurons with elongating neurites and the averages of the longest neurites were calculated. For the experiment of DRG neurons cocultured with siRNA‐applied RSCs, the average neurite length of each neuron was quantified by using the Image J plug‐in AnalyzeSkeleton (2D/3D).^[^ ^82^, ^83^ ^]^

### Neurite Outgrowth Assay—Co‐Culture with SCs

Freshly prepared RSCs were seeded on the 48‐well plate which was coated with PLL and laminin at a density of 2.0 × 10^6^ cells cm^−2^. 3 h later, 1.0 × 10^−4^ DRG neurons cm^−2^ were seeded on the RSCs. To investigate the GFRα1 function on RSCs, a GFRα1 neutralizing antibody (GFRα1 Neut. Ab, 10 µg mL^−1^, R&D Systems, Minneapolis, MN)^[^ ^84^, ^85^ ^]^ was added to the DRG medium. As a control, Goat IgG antibody (10 µg mL^−1^, R&D Systems) was added (n = 3 per group). 48 h later, cells were fixed with 4% PFA.

### Neurite Outgrowth Assay—Culture with Conditioned Medium with RSCs

Culture medium for RSCs was replaced with DRG medium 1 day after seeding RSCs on the 48‐well plate. The next day, the culture medium was centrifuged at 3000 g for 10 min, and the supernatant was used for DRG culture seeded on the 48‐well plate coated with PLL and laminin. 48 h later, plates were fixed with 4% PFA. To inhibit the function of GFRα1 in the culture medium of RSCs, GFRα1 Neut. Ab (10 µg mL^−1^, R&D Systems) was added to the DRG medium. Controls were DRG culture with or without GFRα1 Neut. Ab.

### Neurite Outgrowth Assay—Knock Down of GFRα1 in SCs

Expression of GFRα1 in RSCs was knocked down by an application of siRNA (Accell rat Gfra1 25454, Horizon Discover, UK) with the provided protocol. As a control, non‐targeting siRNA (Horizon Discover, UK) and no treated RSCs were used. In brief, freshly prepared RSCs were cultured on 48 well plates, followed by changing a media containing siRNA the next day. To confirm the decrease of the GFRα1expression, Western blotting of RSCs was performed 3 days later. For co‐culture of knocked down RSCs with DRG neurons, RSCs were washed with PBS twice 2 days after siRNA administration, followed by seeding of freshly prepared DRG neurons on RSCs. Plates were fixed with 4% PFA 48 h later.

### Neurite Outgrowth Assay—GFRα1 Administration

For analyzing the effect of soluble GFRα1 (sGFRα1), rat GFRα1 protein (10 µg mL^−1^, R&D Systems) was added to the DRG media after seeding DRG neurons. As a control for GFRα1, Human IgG (10 µg mL^−1^, R&D Systems) was added (n = 3 per group). For analyzing the effect of immobilized GFRα1 (iGFRα1), the culture plate was incubated with rat GFRα1 protein solution (10 µg mL^−1^ in PBS) or control protein (human IgG protein, 10 µg mL^−1^, R&D Systems) for 3 h at 37°, before seeding DRG neurons. 48 h later, neurons were fixed with 4% PFA.

### Neurite Outgrowth Assay—GDNF Blocking

To confirm the blocking of GDNF by GDNF Neut. Ab (10 µg mL^−1^, goat IgG, R&D Systems), rat GDNF protein (2 µg mL^−1^, PeproTech), and GDNF Neut. Ab were added after seeding DRG neurons (Figure S1). To investigate the GDNF independence, GDNF Neut. Ab was added to the DRG media with iGFRα1. As a control for GDNF protein and GDNF Neut. Ab, Human IgG (2 µg mL^−1^, R&D Systems) and goat IgG (10 µg mL^−1^, R&D Systems) were used (n = 3 per group).

### Neurite Outgrowth Assay—GDNF Coating on iGFRα1

To analyze the effect of GDNF coating (iGDNF) on iGFRα1, GDNF (2 µg mL^−1^, Pepro Tech) was incubated for 3 h after iGFRα1 coating. As a control for iGDNF, human IgG protein was used (n = 3 per group).

### Neurite Outgrowth Assay—NCAM Blocking

To investigate the NCAM dependency, an NCAM neutralizing antibody (10 µg mL^−1^, rabbit IgG, Merck)^[^ ^86^, ^87^ ^]^ was added to the DRG media. As a control, rabbit IgG (10 µg mL^−1^, R&D Systems) was used (n = 3 per group).

### Neurite Outgrowth Assay—Integrinβ1 Blocking

To investigate the integrinβ1 dependency, an integrinβ1 neutralizing antibody (10 µg mL^−1^, HMβ1‐1, Armenian hamster IgG, BioLegend, San Diego, CA, United States)^[^ ^88^ ^]^ was added to the DRG media. As a control, goat IgG (10 µg mL^−1^, R&D Systems) was used (n = 3 per group).

### Surgical Procedures—Crush Injury

Anesthetized rats were placed in a prone position, and a longitudinal 3 cm incision was applied on the right thigh to expose the sciatic nerve. A crush injury was made just distal to the sciatic notch with micro‐mosquito forceps (Fine Science Tools, No. 13010–12, Canada).

### Surgical Procedures—Local Administration

Reagents were injected at 5 mm and 15 mm distal to the crush site through a 34‐gauge needle with a NanoFil syringe (World Precision Instruments, Sarasota, FI, United States). For GFRα1 blocking, GFRα1 Neut. Ab. (total 6 µL, 1 µg/1 µL in PBS) was injected twice at the time of injury and 7 days after injury. As a control, goat IgG (total 6 µL, 6 µg/6 µL PBS) was injected (n = 3 per group). Two weeks after injury, rats were perfused. For GFRα1 administration for axon regeneration, GFRα1(total 4 µL, 1 µg/1 µL in PBS) and GDNF Neut. Ab (total 4 µL, 1 µg/1 µL in PBS) was injected immediately after injury. As a control, human IgG protein and goat IgG antibody were used (n = 3 per group). One week after injury, rats were perfused. For GFRα1 administration for functional recovery, GFRα1(total 4 µL, 1 µg/1 µL in PBS) was injected twice at the time of injury and 7 days after injury. As a control, human IgG proteins were used (n = 9 per group). Eight weeks after injury, subjects were perfused.

### Histology—Immunofluorescent Labeling

Cultured cells were fixed with 4% PFA in 0.1 M PB for 15min. Subjects were perfused with 4% PFA in 0.1 m PB. Then, dissected nerves were fixed overnight with 4% PFA at 4 °C, followed by cryoprotection in 30% sucrose in 0.1 m PB until sectioning. Nerves were sectioned by cryostat at 10 µm intervals and directly mounted on 8 slides in order. Each slide had 4 sciatic nerve sections. Cells and sections were blocked with Tris‐buffered saline (TBS) containing 5% horse serum (Thermo Fisher Scientific) and 0.125% Triton X (Sigma–Aldrich), followed by overnight incubation with primary antibodies at 4 °C. Primary antibodies used in the current study were S100β (1:200, rabbit from Abcam, Cambridge, United Kingdom), pan neurofilament (pNF, 1:1000, mouse from BioLegend, San Diego, CA, United States), β‐III tubulin (1:1000, rabbit from Covance, Princeton, NJ, United States), GFRα1 (1:1000, goat from R&D Systems), RET (1:200, rabbit from Invitrogen), NCAM (1:500, rabbit from GeneTex, Irvine, CA, USA), Integrin β1 (1:200, goat from R&D Systems), integrin α7 (1:200, rabbit from Thermo Fisher Scientific). After washing 3 times with TBS, cells, and sections were incubated with Alexa fluorochrome‐conjugated donkey secondary antibodies (1:1000, Jackson ImmunoResearch, West Grove, PA, USA), and DAPI (100 ng mL^−1^, Sigma–Aldrich) for 1 h at room temperature. Images were taken using an all‐in‐one microscope (Keyence BZ‐X800, Osaka, Japan) or a confocal laser microscope (Olympus FV‐1200, Tokyo, Japan).

### Histology—Toluidine Blue Labeling

For evaluation of remyelinating axons, rats were perfused with 4% PFA, followed by 2.5% glutaraldehyde (TAAB, Berks, England). The most distal portion of the sciatic nerves was embedded in epoxy resin and sectioned axially at 1 µm, followed by staining with toluidine blue (Wako, Osaka, Japan). Images were taken using an all‐in‐one microscope (Keyence BZ‐X800).

### Quantification of Axons

Quantification of axons was performed as described previously.^[^ ^66^ ^]^ Briefly, three consecutive sections on the same slide from the middle part of the nerve were used. Lines perpendicular to sections were set at the quantification points, and axons crossing each line were counted. For normalization, the sum of the axon numbers in the three sections was divided by the sum of the length of each line as the axon density. To calculate the percentage of axon regeneration, the axon density at each point was divided by the density at the uninjured site, which was 1.5 mm proximal to the injury site. To quantify the number of myelinated axons, a 50 µm wide rectangular region was set to cross the center of both the tibial and peroneal parts of the sciatic nerve, and the number of myelinated axons was counted, and divided by the region area to calculate the density of myelinating axons per square millimeter. All quantification was performed blindly by concealing group identity.

### Pull‐Down

Pull‐down of histidine(his)‐tagged rat GFRα1 was performed using Dynabeads his‐tag isolation & pulldown (Thermo Fisher Scientific) as described in the provided protocol. In brief, DRG neurons were cultured overnight, followed by stimulation with his‐tagged rat GFRα1 as described above for 1 h. Then, after 3 times washing with PBS, the whole protein was extracted with lysis buffer and sonication. Lysis buffer consisted of 50 mm sodium‐phosphate, 1% TritonX‐100, 300 mm NaCl, and 0.01% Tween‐20 (Sigma–Aldrich) at pH 8.0. Then, the lysate was mixed with magnetic beads, incubated at room temperature for 5 min, and washed 4 times with the washing buffer consisting of 100 mm sodium‐phosphate, 300 mm NaCl, and 0.01% Tween‐20 at pH 8.0. His‐tagged GFRα1 was eluted by adding the buffer consisting of 300 mm imidazole, 50 mm sodium‐phosphate, 300 mm NaCl, and 0.01% Tween‐20 at pH 8.0 to the beads.

### Western Blotting

To analyze the protein expression of cultured SCs and explore cell signaling of cultured DRG neurons, Western blotting was performed. DRG neurons were stimulated with iGFRα1 as described above and GDNF (2 µg mL^−1^) for 24 h. A whole protein of cultured cells was extracted by applying a lysis buffer, followed by separation with sodium dodecyl sulfate‐polyacrylamide gel electrophoresis and transfer to a polyvinylidene fluoride membrane (Immobilon‐P Membrane; Merck, Darmstadt, Germany). Then, membranes were blocked with 5% powdered milk (<1% fat) in PBS for 1 h at room temperature, followed by overnight incubation with primary antibodies: anti‐GFRα1 (1:1000, goat from R&D Systems), anti‐GDNF (1:1000, goat from R&D Systems), anti‐RET (1:1000, rabbit from Thermo Fisher Scientific), NCAM (1:1000, rabbit from GeneTex), integrin β1 (1:1000, goat from R&D Systems), integrin α7 (1:1000, rabbit from Thermo Fisher Scientific). anti‐FAK (1:1000 from sheep, R&D Systems), anti‐FAK (Phospho‐Tyr397)(1:1000 from rabbit, Signalway Antibody), anti‐Fyn (1:1000 from mouse, R&D Systems), anti‐Fyn (Phospho‐Tyr530)(1:1000 from rabbit, Signalway Antibody), anti‐PI3 kinase (1:500 from rabbit, BioVision), anti‐PI3K (Phospho‐Tyr607)(1:1000 from rabbit, Thermo Fisher), anti‐ERK1/ERK2 (1:1000 from mouse, R&D Systems), anti‐ERK1/ERK2 (Phospho‐Thr202/Tyr204)(1:1000 from rabbit, Cell Signaling), and GAPDH (1:1000, rabbit from Affinity Bioscience). After washing, the membrane was incubated with horseradish peroxidase‐conjugated secondary antibody (1:1000, Novus Biologicals) for 1 h at room temperature. The bands were visualized using Ez WestLumi Plus (ATTO, Tokyo, Japan) and Quantity One v. 4.6.9 (Bio‐Rad) software. To improve the sensitivity of Western blotting of pull‐downed protein for detecting NCAM and integrin α7β1, biotinylated secondary antibodies (1:3000, Jackson ImmunoResearch) were used as a secondary antibody, followed by the incubation with avidin/biotin ABC HRP complex (1:50, Vectastatin ABC‐HRP Kit, Vector Laboratories, CA, USA). Quantification of bands was performed using Image J, as described previously.^[^ ^89^ ^]^

### Behavioral Analysis

Adult female LEWIS rats (8–12 weeks old) were used, and all subjects were habituated for a week before functional evaluation. All analysis was performed blindly. Gait and sensory analysis were conducted at pre‐injury, 4 and 8 weeks after injury. Electrophysiology was performed at 8 weeks after injury.

### Behavioral Analysis—Gait Function

Treadmill gait analysis was performed using the DigiGait system (Mouse Specifics, Inc., Quincy, MA, USA) as described previously.^[^ ^90^ ^]^ The treadmill started at a speed of 10 cm s^−1^ and its speed was increased to 20 cm s^−1^ unless subjects failed to run. The gait videos were analyzed using the DigiGait Imaging and Analysis software 12.2 (Mouse Specifics). From the obtained footprint images, paw length, and paw width were measured, and SFI was calculated with reference to the previous report.^[^ ^91^ ^]^ Five trials were performed, and the average of three trials was divided by the value at pre‐injury.

### Behavioral Analysis—Sensory Function

Sensory function was evaluated by the electric Von Frey test and the Hargreaves test as described previously.^[^ ^92^, ^93^, ^94^ ^]^ Mechanical thresholds of the hind paw were measured using a Dynamic Planter Aesthesiometer (Ugo Vasil, Varase, Italy). Von Frey filaments with bending forces of 0–50 g were applied on the left mid‐planter surface of the hind paw at a rate of 2.5 g s^−1^. When a withdrawal response was observed, the duration from the stimulus to the withdrawal of the paw was recorded. Five trials were performed for each paw, and the average time was divided by the value at pre‐injury. Thermal thresholds in the hind paw were evaluated using a Hargreaves device (Ugo Basile, Varese, Italy). Heat stimulus was applied on the paw for 10s from 35 to 70 °C in intervals of 2.5 °C until withdrawal response of the hind paw was observed. The average time of the five trials was divided by the value at pre‐injury.

### Behavioral Analysis—Electrophysiology

Electrophysiological analysis on the injured nerve was performed using Neuropack MEB‐9102 (NihonKohden, Tokyo, Japan).^[^ ^95^ ^]^ Under general anesthesia, bipolar stimulating electrodes were attached proximal to the injury site, and the compound muscle action potential and terminal latency were recorded by electrodes inserted into the tibialis anterior muscle. Latency, nerve conduction velocity (NCV), and amplitude were measured.

### Behavioral Analysis—Muscle Weight

After the perfusion of the rat, the tibialis anterior muscle and the gastrocnemius muscle were dissected at the proximal and distal bone attachment sites, and their weights were measured.

### Statistics

Multiple‐group comparisons were made using the one‐way analysis of variance (ANOVA) with the Tukey–Kramer test or two‐way repeated measure ANOVA with Bonferroni's test, and two‐group comparisons were made using the unpaired two‐tailed Student's t‐test. All analyses were performed with the JMP software (SAS, Cary, NC, USA) with a pre‐specified significance level of 95%. Data are presented as the mean ± standard error of the mean (SEM).

### Data and Materials Availability

The datasets generated during and/or analyzed during the current study are available from the corresponding author upon reasonable request.

## Conflict of Interest

The authors declare no conflict of interest.

## Author Contributions

T.S. and K.K. performed conceptualization. T.E., M.Y., M.W. did methodology. T.S., T.E., and K.K. did investigation. T.S. and K.K. performed funding acquisition. K.K. did project administration. N.I. performed the supervision. T.S. and K.K. performed writing.

## Supporting information



Supporting Information

## Data Availability

The data that support the findings of this study are available from the corresponding author upon reasonable request.
