# Supplementary material for: GFRα1 Promotes Axon Regeneration after Peripheral Nerve Injury by Functioning as a Ligand
Source: Adv Sci (Weinh). 2024 Dec 4;12(4):2400812. doi: 10.1002/advs.202400812 (PMC11775530; doi:10.1002/advs.202400812)
Supplement: Supplementary file 1 — Supporting Information [file ADVS-12-2400812-s001.pdf]

ADVANCED SCIENCE
Open Access

Supporting Information

for *Adv. Sci.*, DOI 10.1002/advs.202400812

GFRα1 Promotes Axon Regeneration after Peripheral Nerve Injury by Functioning as a Ligand

Tomoaki Suzuki, Ken Kadoya, Takeshi Endo, Miwako Yamasaki, Masahiko Watanabe and Norimasa Iwasaki*

Figure S1

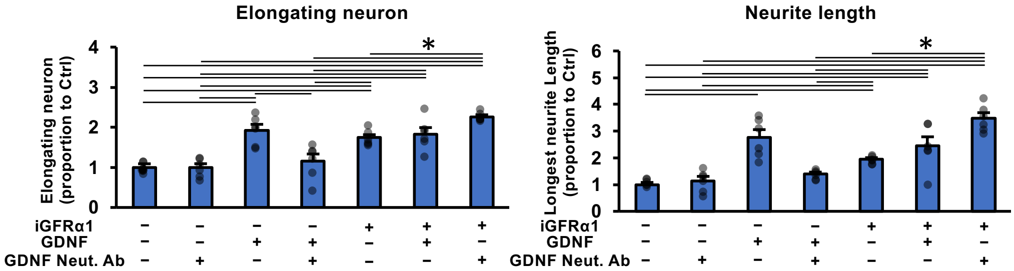

Elongating neuron
Elongating neuron (proportion to Ctrl)
0
1
2
3
4
*
iGFRα1
GDNF
GDNF Neut. Ab
Neurite length
Longest neurite Length (proportion to Ctrl)
0
1
2
3
4
5
6
*
iGFRα1
GDNF
GDNF Neut. Ab
